# Nanoporous Stainless Steel Materials for Body Implants—Review of Synthesizing Procedures

**DOI:** 10.3390/nano12172924

**Published:** 2022-08-25

**Authors:** Metka Benčina, Ita Junkar, Alenka Vesel, Miran Mozetič, Aleš Iglič

**Affiliations:** 1 Department of Surface Engineering, Jožef Stefan Institute, Jamova Cesta 39, 1000 Ljubljana, Slovenia; 2 Laboratory of Physics, Faculty of Electrical Engineering, University of Ljubljana, 1000 Ljubljana, Slovenia

**Keywords:** stainless steel, anodisation, nanoporous morphology

## Abstract

Despite the inadequate biocompatibility, medical-grade stainless steel materials have been used as body implants for decades. The desired biological response of surfaces to specific applications in the body is a highly challenging task, and usually not all the requirements of a biomaterial can be achieved. In recent years, nanostructured surfaces have shown intriguing results as cell selectivity can be achieved by specific surface nanofeatures. Nanoporous structures can be fabricated by anodic oxidation, which has been widely studied for titanium and its alloys, while no systematic studies are so far available for stainless steel (SS) materials. This paper reviews the current state of the art in the anodisation of SS; correlations between the parameters of anodic oxidation and the surface morphology are drawn. The results reported by various authors are scattered because of a variety of experimental configurations. A linear correlation between the pores’ diameter anodisation voltage was deduced, while no correlation with other processing parameters was found obvious. The analyses of available data indicated a lack of systematic experiments, which are recommended to understand the kinetics of pore formation and develop techniques for optimal biocompatibility of stainless steel.

## 1. Introduction

Many body implants, such as orthopaedic, cardiovascular and dental, are made from stainless steel. The most common types of stainless steel used in biomedical applications are 316, 316L and 304 [1,2,3,4]. The main difference between the 316 and 304 grades is that 316 grade contains about 2–3 percent molybdenum [4]. The 316L grade of stainless steel has a lower amount of carbon than 316 grade. Stainless steel of medical grade is characterised by excellent mechanical properties and reasonable chemical inertness. The biocompatibility, however, is often inadequate because of the structure and composition. The rather poor biocompatibility in the case of vascular stents is reflected in thrombogenic response, uncontrolled proliferation of smooth muscle cells and a poor proliferation of endothelial cells, while in the case of orthopaedic devices, poor adhesion of osteoblast cells and a high risk of bacterial infections. To overcome these issues, various coating procedures were developed, which showed more or less success. Deposition of a thin film of highly biocompatible material, such as some types of ceramics, biocompatible polymers, composites and matrixes that incorporate drug releasing agents, has been proposed [5,6,7,8,9], but issues regarding long-term stability, mainly due to the discrepancies between mechanical properties as well as poor adhesion and gradual changes of the interface, still persist. Furthermore, stainless steel contains traces of nickel, which is regarded as an allergenic and cytotoxic material [10,11,12]. The release of nickel from the stainless-steel implants is minimal but, in some cases, sufficiently large to cause serious health problems. It is known that the biocompatibility of metal oxide coatings is often better than the parent metals used as substrates because the oxides usually suppress the release of toxic ions [13,14]. On the other hand, it is also known that the oxidation of stainless steels or similar materials leads to the formation of various Fe and Cr oxides, and not all of them are considered biocompatible. Furthermore, the oxide film is often not compact enough to prevent diffusion of any ions from the bulk stainless steel onto the oxide surface and thus the release of ions. Many authors suggested chromium oxide as the most biocompatible oxide, which might be formed on the stainless steel surface. The traditional method for oxidation of stainless steel, i.e., thermal treatment in an oxygen-rich atmosphere, has been studied in detail for decades, and the general conclusion is that low-temperature treatment favours the formation of iron oxides with a significant amount of nickel, while oxidation at elevated temperatures will lead to the formation of a film predominantly composed of chromium oxide [15]. The iron oxide film formed at lower temperatures, however, usually persists on the surface of stainless steel oxidised at higher temperatures. The film formed at the high temperature is therefore not pure and compact chromium oxide. The biocompatibility of oxides formed on the surface of implants strongly depends on their type, thickness and quality. Corrosion resistance, ion release and mechanical stability of formed oxides on the surface should also be considered to prevent undesired infections or failures of medical implants. In addition, nanostructured oxide surfaces could offer additional benefits in terms of cell adhesion and selectivity, as it is already a well-known fact that cells react differently to specific surface nanofeatures [16,17]. There are alternative methods of oxidation using non-equilibrium techniques such as gaseous plasma, which may also enable surface nanostructuring, but they may not be practical in all applications. Yet another alternative is oxidation by electrochemical methods. This particular method has been probed for various metal materials, which showed significant improvement in biocompatibility not only due to the formation of oxide layers but also due to altered surface nanotopography [16,18,19,20]. Nanostructured surfaces with specific surface topography dictate cell–surface interactions and could be a powerful tool for optimising cell adhesion as well as promoting the growth of one cell type over another. This paper aims to review the most important literature and draw correlations between the processing parameters and the surface finish.

## 2. Literature Survey

Electrochemical oxidation of metals and alloys is an established technique nowadays used in mass production. The basic concept is to dip the workpiece into an ionic solution and apply a positive charge. The workpiece acts as the anode, while the container is usually grounded and therefore acts as the cathode. Alternatively, the container is made from a dielectric, and a counter electrode is immersed in the bath. The technique is usually referred to as anodic oxidation or anodisation. Probably the best-known technique is anodisation of titanium, used for the formation of nanotubular or nanoporous films [18,21] and anodisation of aluminium, which is used for the formation of a thin oxide film [22,23,24], which prevents any further degradation of the material.

The nanoporous structure of metals and their alloys for biomedical applications can also be prepared by other techniques such as non-thermal plasma [25,26]. Although metals, such as titanium, aluminium and stainless steel, spontaneously form a thin oxide film in a harsh environment, anodisation is commonly applied in order to enhance corrosion resistance or biocompatibility of the metallic materials. The reason for using anodisation for oxidation of the stainless steel is twofold: (i) ability to control the composition of the oxide film and (ii) various morphologies of the oxide film on the nano-scale, which, for instance, influence the response of biological material in contact with the anodised surfaces.

**Figure 1:**
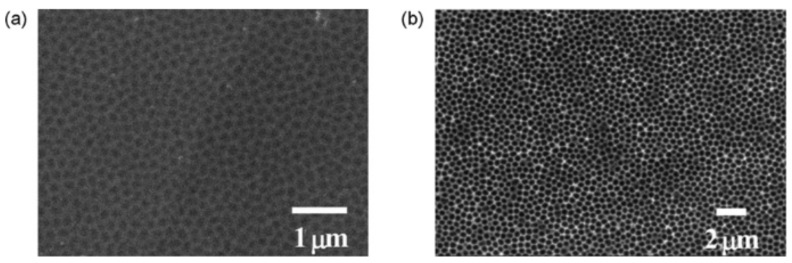
SEM images of nanoporous 316SS surfaces formed at 60 V in perchloric acid containing organic solvents: (**a**) ethanol, (**b**) acetic acid. Reprinted from Ref. [27] with permission from Elsevier.

As early as 2012, Tsuchiya et al. [27] reported the spontaneous formation of pores on type 316 stainless steel upon electrochemical oxidation in an ionic liquid containing different organic solvents. The samples were first polished using three different techniques and cleaned well by chemical methods. They were carefully dried in a nitrogen stream to ensure minimal surface contamination. Anodisation was performed with different organic solvents containing perchlorate ions. The cathode was a platinum electrode. The anodisation voltage was slowly increased until the final voltage was achieved. The samples treated at various experimental conditions were briefly rinsed and dried and then characterised by scanning electron microscopy (SEM) and atomic force microscopy (AFM). These techniques were used to estimate the surface morphology and determine the lateral dimensions as well as the depth of the morphological features. By using 10 vol. % of perchloric acid and 90% of ethylene glycol, the authors reported increasing diameters of the pores formed upon anodisation at increasing voltages. At the voltage of 20 V, the pore diameter was below 100 nm, but then it increased significantly until the pores of 345 nm diameters were obtained at the voltage of 60 V. When the ethylene glycol was replaced by ethanol or acetic acid, the authors used only the voltage of 60 V and observed nanopores with a diameter of about 100 nm and 300 nm, respectively. By using acetic acid in the electrolyte, the nanopores were more evenly distributed and of almost uniform diameter (Figure 1).

Tsuchiya et al. [27] also studied the effect of water in an organic solvent in the anodisation vessel and found that small quantities caused much faster pore formation. When using an electrolyte containing water and ethylene glycol, the diameter of nanopores was increased (about 300 nm) compared to the diameter of nanopores obtained in a water-free ethylene glycol-based electrolyte (about 150 nm). The results were explained by a much larger electrical current in cases where water was present in the electrolyte.

The biological response of the anodised nanostructured (nanotubular and nanoporous) titanium surfaces has been immensely studied [18,21,28] The biological response of the nanoporous stainless steel was elaborated by Pan et al. [29]. Different nanopores were fabricated on 316L stainless steel by anodisation. They used perchloric acid and ethylene glycol monobutylether as the electrolyte and studied the evolution of the surface morphology. Unlike Tsuchiya et al. [27], who performed anodisation at room temperature, Pan et al. [29] selected temperatures between 5–10 °C. At the anodisation voltage of 30 V, they found the diameter of nanopores was about 40 nm, roughly three times smaller than Tsuchiya et al. [27]. The next voltage probed was 45 V, and the pore diameter was 75 nm. This is significantly smaller than what was observed by Tsuchiya et al. [27] at a voltage of 40 V. The discrepancy is difficult to explain only from the anodisation parameters, but it should be stressed that Pan et al. [29] did not use pure ethylene glycol but rather ethylene glycol monobutylether. Furthermore, the treatment times adopted by Pan et al. [29] were much longer, at about half an hour. Pan et al. [29] managed to synthesise top-quality nanopores in a broad range of diameters from about 40 nm to 210 nm. They also determined the mean roughness as deduced from AFM measurements. The proliferation of fibroblast cells was studied on samples of various pore diameters. The best results in terms of enhanced cell proliferation were observed for nanopores of small diameters, between about 40 nm and 80 nm.

The performance and behaviour of human cells in response to the different nanotopographies have already been shown previously. It has been shown that, typically, small nanofeatures (raised above 10 nm but below 20 nm high) increase cell adhesion, size and spreading of, for example, endothelia, fibroblasts and mesenchymal stem cells, but larger nanofeatures (app. 100 nm high) generally inhibit cell spreading, cytoskeletal organization and functional differentiation [30,31,32,33,34]. Initial interaction between cells and nanotopography is through the filopodia, which are fine cell membrane projections that collect the nanotopographical information from the materials surface [34].

Similar experiments as in the pioneering work of Tsuchiya et al. [27] were also performed by Ni et al. [35]. They used exactly the same electrolyte, but the concentration of the perchloric acid was only 5.3 vol. %. Unfortunately, Ni et al. [35] did not report the treatment time nor the electrolyte temperature. They probed four voltages, i.e., 20 V, 30 V, 40 V and 50 V. At a voltage of 20 V, no pores were observed by SEM. Interestingly enough, the voltage of 30 V enabled the formation of a uniform nanoporous honeycomb morphology with an average pore diameter of 25 nm only. At a voltage of 40 V, they observed pores of a 50 nm diameter, and at the maximal voltage used in this work of 50 V, they obtained the pores of a diameter of 60 nm. Ni et al. [35]., therefore, reported much smaller pore diameters than Tsuchiya et al. [27] and Pan et al. [29]. Almost identical results were reported by the same group almost simultaneously in the proceedings [36].

Tsuchiya [37] published another paper two years after his report about the formation of nanoporous 316 stainless steel [27]. The experimental conditions were similar to those in his paper [27], except that he also used sodium perchlorate, not only perchloric acid-containing electrolyte. He concentrated on rather large voltages of 50 V or 60 V. At a voltage of 50 V, Tsuchiya obtained ordered pore arrays with an average diameter of about 210 nm. He also estimated the depth of the pores from AFM line profiles and found an average depth of about 15 nm. Other experiments were performed at a voltage of 60 V. When only ethylene glycol and sodium perchlorate were used, he found a pore diameter of about 260 nm, which is not far away from the results reported in [27]. When about 5 vol. % water was added, the average pore diameter increased to 330 nm, and when the water concentration was 12 vol. %, the diameter slightly increased to 350 nm. Interestingly enough, when the water content was 20 vol. %, Tsuchiya [37] did not observe any pores. In conclusion, Tsuchiya [37] mentioned that this technique could also be applied to other metallic substrates, in particular, Inconel 600 and Co–Cr–Mo alloys, and supported the statement with two images of porous surface finishes. Despite the large dimension of the pores, Tsuchiya [37] reported increased osteoblast-like cell proliferation. The results are not sound with the work of Pan et al. [29] and Khaw et al. [38], who found such large pores obsolete in terms of cell proliferation.

Lu et al. [39] also performed the synthesis on stainless steel type 904L. Several different voltages were used. At the voltage of 30 V, the authors measured the current density versus treatment time at different temperatures of the electrolyte from 4 °C to 30 °C. The initial current density significantly depended on the electrolyte temperature. The current densities were about 0.15 A/cm^2^, 0.3 A/cm^2^ and 0.5 A/cm^2^, and in terms of temperature, they were 4 °C, 12 °C and 30 °C, respectively. The initial current density decreased significantly (by an order of magnitude) after about 30 s and stabilised at a very low value for the case of 4 °C, whereas the current density at 30 °C remained moderate at a value of about 0.07 A/cm^2^. Lu et al. [39] also acquired SEM images of the samples treated for 10 min at those temperatures and found perfectly ordered nanopores at 4 °C, whereas the nanopores at 30 °C were stochastically distributed and somehow larger.

In addition, Lu et al. [39] performed anodisation of stainless steel 904L at different voltages, but at the same treatment time of 600 s and electrolyte temperature of 4 °C. They found an enormous dependence of the pore diameter on voltage. The pore diameter at 20 V was as small as about 40 nm, and the structures were highly ordered. Increasing the voltage to 30 V resulted in the formation of highly ordered nanopores with an average diameter of about 80 nm. The voltage of 40 V caused rather unordered pores of a diameter of roughly 100–200 nm. All these results were reported for samples treated for 600 s. A sample was also treated for 300 s, and un-ordered surface morphology was observed. Lu et al. [39] also reported that pitting appeared on this type of stainless steel when the electrolyte temperature was about 40 °C.

Unlike previously cited authors, Lu et al. [39] also reported on the structure of the surface film. They used X-ray photoelectron spectroscopy (XPS) to evaluate the composition and chemical bonding at two different anodisation times. The chemical compositions were measured at various depths of 3 nm, 15 nm, 30 nm, 45 nm and 60 nm. They found little difference in the composition at a depth of 3 nm and 15 nm, indicating the formation of a rather compact surface film. The depth profiling enabled estimation of the thickness of the oxide film, and according to XPS results, it was roughly 10 nm. The surface film was rich in chromium and perhaps iron oxides, and the concentration of nickel was close to the detection limit of the XPS. The original nickel content of 904L stainless steel is about 25%, therefore, more than Cr, which is 20%. Obviously, the anodisation enabled the preferential formation of chromium oxide film on the surface. From high-resolution XPS peaks of iron and chromium, the authors managed to conclude that the surface film actually consisted of Cr_2_O_3_ and Fe_2_O_3_. These are the most stable forms of oxides, so one can conclude that anodic oxidation causes complete oxidation, which is also preferential since the composition of the oxide film does not reflect the composition of the alloy.

Top-quality nanopores were also synthesised on SS316L substrates by Ban et al. [40]. They used a dielectric container and a couple of electrodes. The workpiece was the anode, and the cathode was a Pt foil. The samples were first electropolished for 10 min at a temperature of 80 °C using a water solution of phosphoric and sulphuric acids. As-polished substrates were then exposed to anodisation using anhydrous ethylene glycol containing perchloric acid at 5 wt.%. They used various voltages and treatment times in order to find the optimal conditions. They synthesised pores with a diameter of 50 nm using a voltage of 40 V and treatment time of 10 min. Top-quality pores of a diameter of 80 nm were synthesised at the voltage of 50 V and treatment time of 35 min. They tested such materials for antibacterial properties, and they reported outstanding results for the case of *Listeria monocytogenes*.

Namely, a smooth surface enabled the log/CFU concentrations of 3.6 cm^−2^, whereas the concentration was about 1.6 and 1.4 cm^−2^ for the cases of 50 nm and 80 nm pores, respectively. The nanoporous surfaces were therefore found to be very antibacterial (similarly as it was shown in Ref. [41]) as the density of bacteria dropped for over 100-times as compared to smooth surfaces.

Farrag et al. [42] used various voltages, treatment times, and electrolyte temperatures in order to synthesise nanopores on the surface of stainless steel 316L materials. They also provided some characterisation of the materials using XPS and X-ray diffraction spectrometry (XRD). The aim of their study was to probe such materials as a photoanode for visible light photo-electrolysis. They performed anodisation using a mixture of ethylene glycol and 5 vol. % of perchloric acid to obtain highly oriented nanopores with an average diameter of about 120 nm. The concentration of perchloric acid of 3 vol. % or 10 vol. % did not enable the formation of the nanopores. Furthermore, the pores were much smaller at lower voltages; about 40 nm at 30 V and about 70 nm at 35 V. Prolonged treatment times caused degradation of the porous structure, and the same applied to elevated electrolyte temperatures. These authors also probed admixtures of methanol as well as inorganic acids. Such admixtures prevented the formation of ordered nanopores but facilitated an extremely porous surface finish, which is useful for photocatalysis.

Bae et al. [43] synthesised nanoporous 316L stainless steel surfaces to study the platelet adhesion, cellular response and drug delivery capability of such materials. Unlike previously cited authors, Bae et al. [43] used an electrolyte composed of 1 M H_2_SO_4_ and 1.0 wt.% hydrofluoric acid solution with pH 2–3. A stainless steel substrate was the anode, and the cathode was a platinum plate. The anodic oxidation process was performed at room temperature. Such substrates were probed for a biological response using a variety of techniques, including SEM for estimation of the blood platelet adhesion on the surface, a cell migration assay and an in vitro cell proliferation assay. The substrates were also loaded with everolimus, and the drug amount, as well as the release rate, was measured. The surface morphology changed significantly after the electropolishing for 10 min at a voltage of 10 V, an electrolyte temperature of 50 °C and additional anodic oxidation at room temperature. Namely, evenly distributed nanopores of a diameter of about 100 nm were observed both by SEM and AFM. The latter technique also enabled the estimation of the average roughness, which was found to be close to 10 nm. The concentration of blood platelets was decreased by five times as compared to the untreated sample, while the density of smooth muscle cells decreased by about 30%.

The surface morphology of nanoporous stainless steel enabled a small increase in the drug amount, but the release rate dropped significantly as compared to the untreated samples. About 75% of the drug was released on the first day in the case of an untreated sample, but the samples prepared according to the methods developed by Bae et al. [43] enabled much slower release, i.e., 18% till the end of the first day, 40% till day 4 and 62% after a week. The surface finish with dense pores enabled gradual drug release, which was measurable even a month after deposition.

An advanced approach regarding previous attempts of anodisation of stainless steel was disclosed by Rodriguez-Contreras et al. [44]. They used a mixture of sulfuric acid and 30% hydrogen peroxide as the electrolyte. They limited the experiments to 96% of sulfuric acid. The electrolyte was allowed to heat continuously during the anodisation process. The starting temperature was 40 °C, and the ultimate was 80 °C. The ultimate temperature was reached after 12 min of anodisation. Unfortunately, the anodisation voltage was not specified; only the current density of 700 A/m^2^ was disclosed. The treatment caused a rich surface morphology of unevenly distributed semi-circular pores of diameter below 20 nm. High-resolution XPS spectra revealed both chromium oxide (Cr_2_O_3_) as well as hydroxide (Cr(OH)_3_). Several iron oxides were detected, with FeO and Fe_2_O_3_ being the most predominant. The concentration of nickel compounds was also above the detection limit. An outstanding antibacterial property of the samples treated according to the innovative methods was proved for *E. Coli* and *B. Subtilis*. The proliferation of osteoblast cells improved significantly, but the concentration of fibroblast cells did not change significantly as compared to untreated stainless steel samples. Osteogenic cell filopodia morphology was found to be beneficial on the surface of nanostructured anodised samples. Unlike the above-cited authors, Rodriguez-Contreras et al. [44] also reported on the surface energy. The anodisation caused an increased polar component of the surface energy from about 5 to 8–10 mN/m (depending on the type of stainless steel, either 304 or 316), and the static water contact angle decreased from about 78 to 69 and 64 for SS 304 and 316, respectively. The anodisation, therefore, didn’t cause a large change in the surface hydrophilicity. Here, it is worth mentioning that the authors haven’t reported the storage time between the anodisation and the wettability measurements, which may be crucial due to the hydrophobic recovery typical for such materials.

Recently, Wang et al. [45] reported on the optimization of anodising parameters. Rich morphology (randomly arranged pore-like structures) was achieved on the surface of stainless steel 304 by anodisation using an electrolyte composed of nitric acid, sulfuric acid, hydrochloric acid, sodium chloride and urea. Degreasing was performed by treatment with acetone, and the final cleaning step was sonification in ethanol. Both electrodes were made from stainless steel. The samples were additionally treated with acids after the anodisation. The anodisation itself was performed for 1–5 min at a voltage as low as 3–5 V. Numerous experimental parameters were probed. Optimal results in terms of appropriate nanopores were reported for the nitric acid solution containing thiourea and sodium chloride. A specific concentration of all reactants and the anodisation time of 2 min and voltage of 5 V enabled the porosity to be as large as 37%. Such a morphology was found to be beneficial for the adhesion of polymers due to the protruding structures on the surface of SS.

The key processing parameters and results reported by different authors are summarised in Table 1.

## 3. Correlations between the Anodisation Parameters and the Surface Treatment

**Figure 2:**
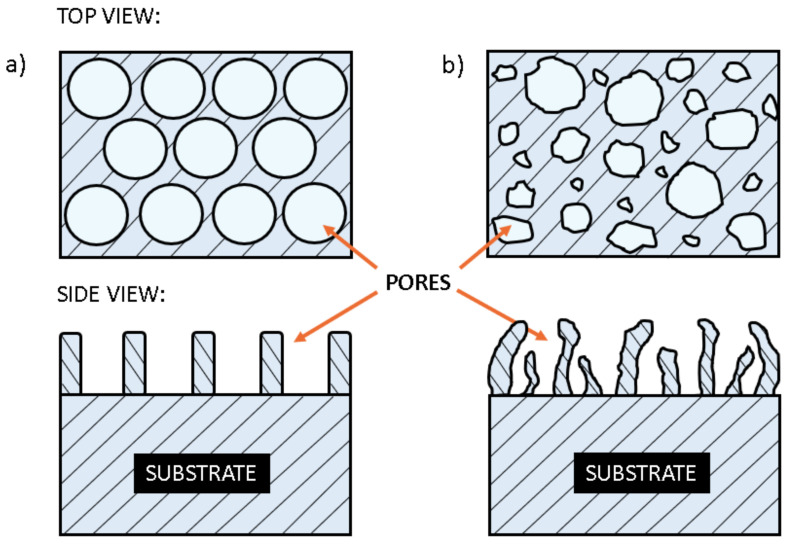
Schematic presentation of the stainless steel surface finish with dense (ordered) nanopores (**a**) and with less oriented structures on the surface (**b**). The lower images side views and the upper images represent the top view.

The literature survey enables correlations between the processing parameters and the surface morphology despite the fact that not all authors presented comprehensive results. As a rough conclusion, one can distinguish between two morphologies: (i) evenly distributed nanopores of almost equal parameters, and (ii) formation of an uneven morphology, which may or may not consist of circular nanopores. The two extremes are illustrated in Figure 2. Figure 2a represents the surface of stainless steel schematically after anodisation that leads to the formation of evenly distributed nanopores. The substrate consists of stoichiometric stainless steel, and the walls between the pores are metal oxides. Such a surface finish was reported by authors who used a large concentration of organic components in the electrolyte. The most frequently used organic component was ethylene glycol. For instance, Tsutchiya et al. [27] were able to prepare ordered pore arrays in ethylene glycol-, di-ethylene glycol- and poly-ethylene glycol-based electrolytes, while Bae et al. [43] reported on the formation of nanoporous structures without ordered morphology by using inorganic electrolyte. The formation of highly oriented structures is only reported for a limited range of processing parameters, which include the concentration of other chemicals in the electrolyte, the electrolyte temperature, the anodisation voltage and the corresponding current density, the treatment time, and perhaps some peculiarities which are not revealed in the literature.

Figure 2b represents the surface finish reported by authors who didn’t use the range of parameters useful for the formation of dense pores of almost equal diameters. The oxide whiskers grow sporadically in such cases, and the final morphology may or may not consist of spherical nanopores. The diameter of the nanopores is usually in a range between, say, 10 and a few 100 nm. Such a surface finish was reported by authors who used electrolytes free from organic chemicals [43,44,45].

**Figure 3:**
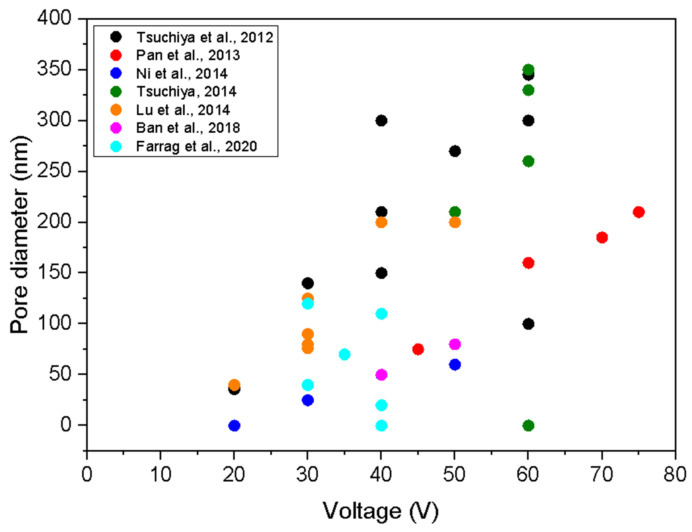
The diameter of nanopores versus the anodisation voltage. Authors and year of publication of the studies stated in the legend are also cited in the references at the end of the manuscript.

The results summarised in Section 2 of this manuscript were further analysed in the present study. Most authors reported the anodisation voltage and the electrolyte temperature. Figure 3 is a plot of the average diameter of nanopores versus the anodisation voltage. The circles represent cases where nanopores are dense and of almost the same diameter, while the ovals are for cases where the surface morphology is richer. In this case, we just present the typical values. For example, if the surface is rich in features of diameter in the range between 20 and 100 nm and only one pore of diameter 500 nm is observed from the SEM/AFM image, we ignore the pore of 500 nm. The measured values are scattered, but the general trend is obvious: the pore diameter increases with increasing voltage.

**Figure 4:**
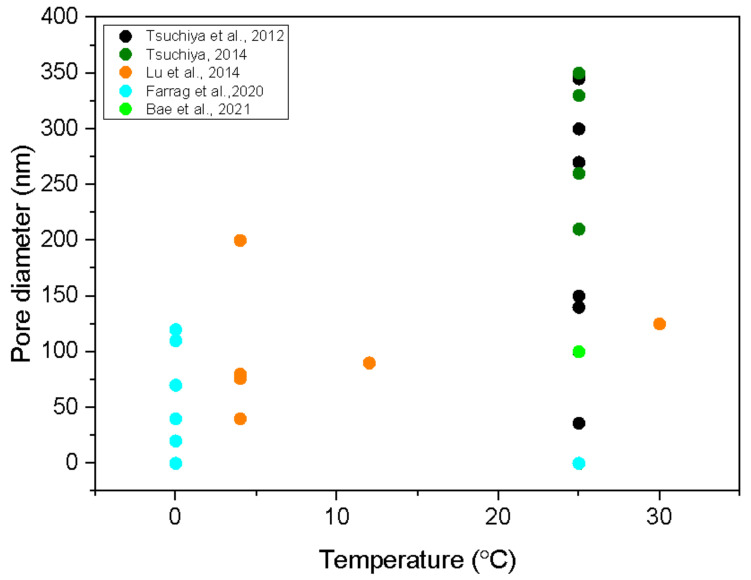
The diameter of nanopores versus the electrolyte temperature. Authors and year of publication of the studies stated in the legend are also cited in the references at the end of the manuscript.

Figure 4 is a plot of pore diameters versus the electrolyte temperature. Most authors stabilised the temperature, but some let the electrolyte heat up upon processing. In the latter case, only the highest temperature is taken into account. Not many temperatures were probed, so the measurement points in Figure 4 are concentrated to a few temperatures. Unfortunately, no author examined the influence of the electrolyte temperature on the pore diameter systematically. The only author who reported such measurements is Lu et al. [39]. They found a rather linear increase in the pore diameter with increasing temperature. The results of other authors, however, do not support the linear dependence. Namely, as clearly revealed from Figure 4, the measured values are scattered so much that one cannot conclude anything about any temperature dependence. This dependence, therefore, remains a scientific challenge and will have to be elaborated on in the future to clarify any temperature dependence of the pore diameter.

**Figure 5:**
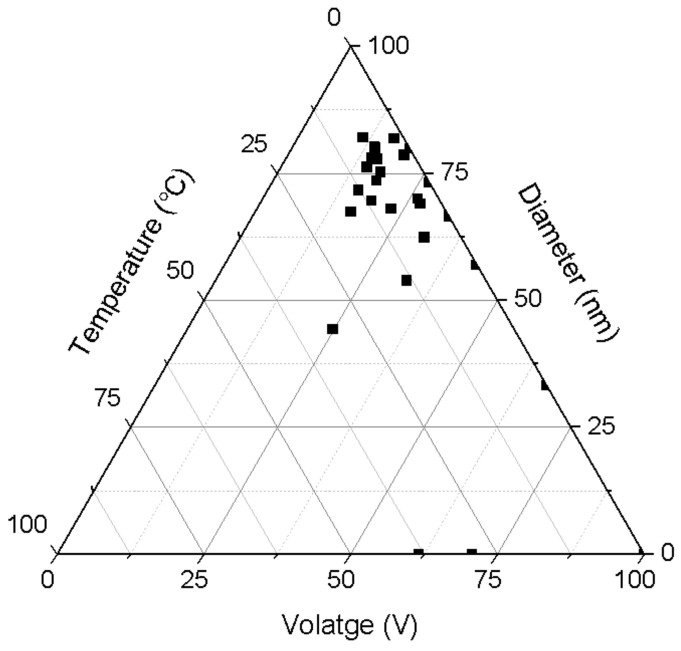
The correlation between the applied voltage, temperature and diameter of nanopores represented as ternary plot.

Temperature and voltage are important parameters that can interdependently influence the pore diameter. Therefore, to see the possible interdependence of both parameters, we plotted the results from Figure 3 and 4 together in Figure 5. In Figure 5 is now more clearly observed what set of temperatures and voltages leads to a certain pore diameter.

To sum up, it is evident that there is a lack of systematic studies of correlations between synthesis parameters, in particular temperature, and SS316L surface morphology. While SS 316L is widely used in biomedicine, it would also be of huge interest to present the effect of different nanopore’s morphologies (e.g., ordered, ununiform) on the biological response (e.g., interaction of SS 316L surface and blood platelets and human cells). Especially when SS316L is used for implants, it is important to consider mechanical stability and corrosion characteristics.

## 4. Conclusions

The scientific literature on the anodisation of stainless steel materials was reviewed and analysed. A large concentration of organic components (preferably between 90 and 95 vol. %) in the electrolyte facilitates the formation of dense nanopores of almost uniform diameter. The depth of the pores was reported by a few authors only, and it is close to 10 nm. The diameter of nanopores depends on various experimental parameters. The only obvious correlation between a processing parameter and the nanopore diameter was found to be the dependence on the applied voltage. In the range of voltages from about 20 to 60 V, the correlation is roughly linear, but the results reported by different authors are scattered significantly. Such a scattering suggests an important role for other processing parameters, but the correlations are not obvious. Most authors performed the anodisation at room temperature, but the reported pore diameters were in the range between a few 10 and several 100 nm, so it seems that the electrolyte temperature may not be the crucial parameter. The science of pore formation is thus still in its infancy. The exact mechanisms and the fine-tuning of the pore morphology, composition and structure remain a scientific challenge. Other properties such as wettability (i.e., the surface energy including the polar and non-polar components) have not been addressed systematically. The surface wettability may play a crucial role in the adhesion of organic drugs as well as body liquids. The biological response was reported by a few authors, and the available literature does not allow for reliable determination of the surface morphology, structure and wettability on the interaction with human blood or cell adhesion and proliferation.

## Figures and Tables

**Table 1:** Review of the experimental parameters and results reported by various authors.

SS Composition	Electrolyte	Time (min)	Voltage (V)	Temperature (°C)	Average Pore Diameter (nm)	Morphology/Pore Distribution	Ref.
316	10 vol. % perchloric acid in ethylene glycol	5	20	25 ^1^	36	Ordered pore arrays.	[27]
	10 vol. % perchloric acid in ethylene glycol	5	30	25	140	Ordered pore arrays.	
	10 vol. % perchloric acid in ethylene glycol	5	40	25	210	Ordered pore arrays.	
	10 vol. % perchloric acid in ethylene glycol	5	50	25	270	Ordered pore arrays.	
	10 vol. % perchloric acid in ethylene glycol	5	60	25	345	Ordered pore arrays.	
	10 vol. % perchloric acid in ethanol	5	60	25	100	Ordered pore arrays.	
	10 vol. % perchloric acid in acetic acid	5	60	25	300	Ordered pore arrays—more defined as with ethanol in the electrolyte.	
	Water-free ethylene glycol containing lithium perchlorate	5	40	25	150	Ordered pore arrays.	
	Water-added ethylene glycol containing lithium perchlorate	5	40	25	300	Ordered pore arrays—more defined as with water-free electrolyte.	
316L	5.3 vol. % perchloric acid in ethylene glycol monobutylether	30	30	5–10	40	Well controlled and highly defined.	[29]
	5.3 vol. % perchloric acid in ethylene glycol monobutylether	30	45	5–10	75	Well controlled and highly defined.	
	5.3 vol. % perchloric acid in ethylene glycol monobutylether	30	60	5–10	160	Well controlled and highly defined.	
	5.3 vol. % perchloric acid in ethylene glycol monobutylether	30	70	5–10	185	Well controlled and highly defined.	
	5.3 vol. % perchloric acid in ethylene glycol monobutylether	30	75	5–10	210	Well controlled and highly defined.	
316L	5 vol. % perchloric acid in ethylene glycol	N/A	20	N/A	0	No pores observed.	[36]
	5 vol. % perchloric acid in ethylene glycol	N/A	30	N/A	25	Uniform nanoporous honeycomb morphology and long-range order.	
	5 vol. % perchloric acid in ethylene glycol	N/A	40	N/A	50	Uniform nanoporous honeycomb morphology and long-range order.	
	5 vol. % perchloric acid in ethylene glycol	N/A	50	N/A	60	Uniform nanoporous honeycomb morphology and long-range order.	
316	10 vol. % perchloric acid in ethylene glycol	5	50	25 ^2^	210	Ordered pore arrays.	[37]
	Ethylene glycol containing sodium perchlorate	N/A	60	25	260	Ordered pore arrays.	
	Ethylene glycol containing sodium perchlorate and water (app. 5 vol. %)	N/A	60	25	330	Ordered pore arrays.	
	Ethylene glycol containing sodium perchlorate and water (app. 12 vol. %)	N/A	60	25	350	Ordered pore arrays.	
	Ethylene glycol containing sodium perchlorate and water (app. 20 vol. %)	N/A	60	25	0	No pores observed.	
316	10 vol.% perchloric acid in ethylene glycol	0.83	50	4	200	Orderly nanopores.	[39]
904L	10 vol. % perchloric acid in ethylene glycol	10	30	4	76	Regular and orderly nanoporous morphology	
	10 vol. % perchloric acid in ethylene glycol	10	30	12	90	Regular and orderly nanoporous morphology	
	10 vol. % perchloric acid in ethylene glycol	10	30	30	125	Regular and orderly nanoporous morphology	
	10 vol.% perchloric acid in ethylene glycol	10	20	4	40	Orderly nanoporpous morphology.	
	10 vol. % perchloric acid in ethylene glycol	10	30	4	80	Orderly nanoporous morphology.	
	10 vol. % perchloric acid in ethylene glycol	10	40	4	200	Less orderly nanoporous morphology as with voltage of 20 V and 30 V.	
	10 vol. % perchloric acid in ethylene glycol	10	50	4	100–200	Unorderly morphology with individual nanopores; nanoporous anodic films almost dissolved.	
	10 vol. % perchloric acid in ethylene glycol	0.83	50	4	100–200	Unorderly morphology with individual nanopores.	
316L	5 wt.% perchloric acid in anhydrous ethylene glycol solution	10	40	N/A	50	Nanoporous surface. Before anodisation the samples were electropolished in a mixture solution of phosphoric acid and sulfuric acid (60%:40% *v*/*v*), at an applied potential of 3.5 V, at 80°C for 10 min.	[40]
		35	50		80		
316	A mixture of equal volumes of H_2_SO_4_ and aqueous H_2_O_2_	N/A	N/A	0 ^3^	17.6 ± 7.1	Nanotopography; crystalline mesoporous layer of oxide on the surface.	[44]
304	A mixture of equal volumes of H_2_SO_4_ and aqueous H_2_O_2_	N/A	N/A	0	16.4 ± 4.2	Nanotopography; crystalline mesoporous layer of oxide on the surface.	
316L	5 vol. % perchloric acid in ethylene glycol	10	30	0 ^4^	120	Regular arrays of surface pores/Self-organized surface nanopores.	[42]
	5 vol. % perchloric acid in ethylene glycol	10	30	0	40	Regular arrays of surface pores/Self-organized surface nanopores.	
	5 vol. % perchloric acid in ethylene glycol	10	35	0	70	Regular arrays of surface pores/Self-organized surface nanopores.	
	5 vol. % perchloric acid in ethylene glycol	10	40	0	110	Regular arrays of surface pores/Self-organized surface nanopores.	
	5 vol. % perchloric acid in ethylene glycol	11	40	0	50–200	Destroyed morphology with individual nanopores.	
	5 vol. % perchloric acid in ethylene glycol	0.33	40	0	20	Partially destroyed morphology.	
	5 vol. % perchloric acid in ethylene glycol	10	40	25	0	Destroyed morphology, no pores observed.	
	3 vol. % perchloric acid in ethylene glycol	10	40	0	0	No pores observed.	
	10 vol. % perchloric acid in ethylene glycol	10	40	0	0	No pores observed.	
304	Nitric acid concentration 90 mL L^−1^, thiourea concentration 3.5 g L^−1^, sodium chloride concentration 20 g L^−1^	2	5.0	N/A	N/A	Nanopore structure with an average porosity of 36.75%.	[45]
316L	1 M H_2_SO_4_ and 1.0 wt.% hydrofluoric acid solutions with a pH of 2–3	N/A	N/A	25 ^5^	100	Nanopores. Before anodisation the samples were electropolished in a H_2_SO_4_, at 50 °C, at a constant voltage of 10 V and 10 A for 10 min.	[43]

^1^ Authors stated: “room temperature”; for the purpose of comparison in this study, 25 °C was considered as the temperature used. ^2^. Authors stated: “room temperature”; for the purpose of comparison in this study, 25 °C was considered as the temperature used. ^3,4^ Authors stated: “ice bath”; for the purpose of comparison in this study, 0 °C was considered as the temperature used. ^5^ Authors stated: “room temperature”; for the purpose of comparison in this study, 25 °C was considered as the temperature used.

## References

[1] Lodhi M., Deen K., Greenlee-Wacker M., Haider W. (2019). Additively manufactured 316L stainless steel with improved corrosion resistance and biological response for biomedical applications. Addit. Manuf..

[2] Thangaraj B., TS Nellaiappan S.N., Kulandaivelu R., Lee M.H., Nishimura T. (2015). A facile method to modify the characteristics and corrosion behavior of 304 stainless steel by surface nanostructuring toward biomedical applications. ACS Appl. Mater. Interfaces.

[3] Andersen P.J. (2020). Stainless steels. Biomaterials Science.

[4] Resnik M., Benčina M., Levičnik E., Rawat N., Iglič A., Junkar I. (2020). Strategies for improving antimicrobial properties of stainless steel. Materials.

[5] Santos A., Aw M.S., Bariana M., Kumeria T., Wang Y., Losic D. (2014). Drug-releasing implants: Current progress, challenges and perspectives. J. Mater. Chem. B.

[6] Lyndon J.A., Boyd B.J., Birbilis N. (2014). Metallic implant drug/device combinations for controlled drug release in orthopaedic applications. J. Control. Release.

[7] Gulati K., Ivanovski S. (2017). Dental implants modified with drug releasing titania nanotubes: Therapeutic potential and developmental challenges. Expert Opin. Drug Deliv..

[8] Barik A., Chakravorty N. (2019). Targeted drug delivery from titanium implants: A review of challenges and approaches. Expert Opin. Drug Deliv..

[9] Kadambi P., Luniya P., Dhatrak P. (2021). Current advancements in polymer/polymer matrix composites for dental implants: A systematic review. Mater. Today Proc..

[10] Biesiekierski A., Wang J., Gepreel M.A.-H., Wen C. (2012). A new look at biomedical Ti-based shape memory alloys. Acta Biomater..

[11] Padiyar N. (2011). Nickel allergy-Is it a cause of concern in everyday dental practice?. Sch. J..

[12] Wataha J.C., Drury J.L., Chung W.O. (2013). Nickel alloys in the oral environment. Expert Opin. Drug Deliv..

[13] Chu C., Wang R., Hu T., Yin L., Pu Y., Lin P., Wu S., Chung C.Y., Yeung K., Chu P.K. (2008). Surface structure and biomedical properties of chemically polished and electropolished NiTi shape memory alloys. Mater. Sci. Eng. C.

[14] Junkar I., Zaplotnik R., Benčina M., Mozetič M. (2020). Method for treatment medical devices made from nickel—Titanium (NiTi) alloys. U.S. patent.

[15] Vesel A., Drenik A., Elersic K., Mozetic M., Kovac J., Gyergyek T., Stockel J., Varju J., Panek R., Balat-Pichelin M. (2014). Oxidation of Inconel 625 superalloy upon treatment with oxygen or hydrogen plasma at high temperature. Appl. Surf. Sci..

[16] Junkar I., Kulkarni M., Benčina M., Kovač J., Mrak-Poljšak K., Lakota K., Sodin-Šemrl S.n., Mozetič M., Iglič A. (2020). Titanium dioxide nanotube Arrays for cardiovascular stent applications. ACS Omega.

[17] Benčina M., Rawat N., Lakota K., Sodin-Šemrl S., Iglič A., Junkar I. (2021). Bio-Performance of Hydrothermally and Plasma-Treated Titanium: The New Generation of Vascular Stents. Int. J. Mol. Sci..

[18] Kulkarni M., Mazare A., Gongadze E., Perutkova Š., Kralj-Iglič V., Milošev I., Schmuki P., Iglič A., Mozetič M. (2015). Titanium nanostructures for biomedical applications. Nanotechnology.

[19] Kulkarni M., Flašker A., Lokar M., Mrak-Poljšak K., Mazare A., Artenjak A., Čučnik S., Kralj S., Velikonja A., Schmuki P. (2015). Binding of plasma proteins to titanium dioxide nanotubes with different diameters. Int. J. Nanotechnol..

[20] Huang H.-H., Wu C.-P., Sun Y.-S., Lee T.-H. (2013). Improvements in the corrosion resistance and biocompatibility of biomedical Ti–6Al–7Nb alloy using an electrochemical anodization treatment. Thin Solid Film..

[21] Lee K., Mazare A., Schmuki P. (2014). One-dimensional titanium dioxide nanomaterials: Nanotubes. Chem. Rev..

[22] Iwai M., Kikuchi T., Suzuki R.O. (2021). High-speed galvanostatic anodizing without oxide burning using a nanodimpled aluminum surface for nanoporous alumina fabrication. Appl. Surf. Sci..

[23] Cao J., Gao Z., Wang C., Muzammal H.M., Wang W., Gu Q., Dong C., Ma H., Wang Y. (2020). Morphology evolution of the anodized tin oxide film during early formation stages at relatively high constant potential. Surf. Coat. Technol..

[24] Suzuki Y., Kawahara K., Kikuchi T., Suzuki R.O., Natsui S. (2019). Corrosion-resistant porous alumina formed via anodizing aluminum in etidronic acid and its pore-sealing behavior in boiling water. J. Electrochem. Soc..

[25] Tucker B.S., Aliakbarshirazi S., Vijayan V.M., Thukkaram M., De Geyter N., Thomas V. (2021). Nonthermal plasma processing for nanostructured biomaterials and tissue engineering scaffolds: A mini review. Curr. Opin. Biomed. Eng..

[26] Benčina M., Junkar I., Zaplotnik R., Valant M., Iglič A., Mozetič M. (2019). Plasma-induced crystallization of TiO_2_ nanotubes. Materials.

[27] Tsuchiya H., Suzumura T., Terada Y., Fujimoto S. (2012). Formation of self-organized pores on type 316 stainless steel in organic solvents. Electrochim. Acta.

[28] Gongadze E., Kabaso D., Bauer S., Slivnik T., Schmuki P., Van Rienen U., Iglič A. (2011). Adhesion of osteoblasts to a nanorough titanium implant surface. Int. J. Nanotechnol..

[29] Pan H.-A., Liang J.-Y., Hung Y.-C., Lee C.-H., Chiou J.-C., Huang G.S. (2013). The spatial and temporal control of cell migration by nanoporous surfaces through the regulation of ERK and integrins in fibroblasts. Biomaterials.

[30] Sjöström T., Dalby M.J., Hart A., Tare R., Oreffo R.O., Su B. (2009). Fabrication of pillar-like titania nanostructures on titanium and their interactions with human skeletal stem cells. Acta Biomater..

[31] Dalby M., Riehle M., Johnstone H., Affrossman S., Curtis A. (2002). In vitro reaction of endothelial cells to polymer demixed nanotopography. Biomaterials.

[32] Dalby M.J., Andar A., Nag A., Affrossman S., Tare R., McFarlane S., Oreffo R.O. (2008). Genomic expression of mesenchymal stem cells to altered nanoscale topographies. J. R. Soc. Interface.

[33] Dalby M., Giannaras D., Riehle M., Gadegaard N., Affrossman S., Curtis A. (2004). Rapid fibroblast adhesion to 27 nm high polymer demixed nano-topography. Biomaterials.

[34] Dalby M.J., Gadegaard N., Oreffo R.O. (2014). Harnessing nanotopography and integrin–matrix interactions to influence stem cell fate. Nat. Mater..

[35] Ni S., Sun L., Ercan B., Liu L., Ziemer K., Webster T.J. (2014). A mechanism for the enhanced attachment and proliferation of fibroblasts on anodized 316L stainless steel with nano-pit arrays. J. Biomed. Mater. Res. Part B Appl. Biomater..

[36] Ni S., Sun L., Ercan B., Liu L., Ziemer K., Webster T.J. (2014). Enhanced Attachment and Proliferation of Fibroblasts on Anodized 316L Stainless Steel with Nano-pit Arrays. MRS Online Proc. Libr. (OPL).

[37] Tsuchiya H. (2014). Formation of self-organized pore arrays on metallic substrates by anodization and their applications. Mater. Sci. Forum.

[38] Khaw J.S., Bowen C.R., Cartmell S.H. (2020). Effect of TiO_2_ Nanotube Pore Diameter on Human Mesenchymal Stem Cells and Human Osteoblasts. Nanomaterials.

[39] Lu W., Zou D., Han Y., Liu R., Tian C. (2014). Self-organised nanoporous anodic films on superaustenitic stainless steel. Mater. Res. Innov..

[40] Ban G.-H., Rungraeng N., Li Y., Jun S. (2018). Nanoporous stainless steel surfaces for anti-bacterial adhesion performances. Trans. ASABE.

[41] Narendrakumar K., Kulkarni M., Addison O., Mazare A., Junkar I., Schmuki P., Sammons R., Iglič A. (2015). Adherence of oral streptococci to nanostructured titanium surfaces. Dent. Mater..

[42] Farrag H.H., Sayed S.Y., Allam N.K., Mohammad A.M. (2020). Emerging nanoporous anodized stainless steel for hydrogen production from solar water splitting. J. Clean. Prod..

[43] Bae I., Lim K.-S., Park J.-K., Song J.H., Oh S.-H., Kim J.-W., Zhang Z., Park C., Koh J.-T. (2021). Evaluation of cellular response and drug delivery efficacy of nanoporous stainless steel material. Biomater. Res..

[44] Rodriguez-Contreras A., Bello D.G., Flynn S., Variola F., Wuest J.D., Nanci A. (2018). Chemical nanocavitation of surfaces to enhance the utility of stainless steel as a medical material. Colloids Surf. B Biointerfaces.

[45] Wang Y., Guo R., Zhou X., Hu G. (2020). Experimental investigation on optimal anodising parameters of nanopore preparation process on the stainless steel surface. Corros. Eng. Sci. Technol..

